# A superior P-H phosphonite: Asymmetric allylic substitutions with fenchol-based palladium catalysts

**DOI:** 10.1186/1860-5397-2-7

**Published:** 2006-03-30

**Authors:** Bernd Goldfuss, Thomas Löschmann, Tina Kop-Weiershausen, Jörg Neudörfl, Frank Rominger

**Affiliations:** 1 Institut für Organische Chemie, Universität zu Köln, Greinstraße 4, D-50939 Köln, Germany; 2 Dottikon Exclusive Synthesis, Hembrunnstrasse 17, CH-5605 Dottikon, Switzerland; 3 X-ray analyses; 4 Organisch-Chemisches Institut der Universität Heidelberg, Im Neuenheimer Feld 270, D-69120 Heidelberg, Germany

## Abstract

The fenchol-based P-H phosphonite BIFOP-H exeeds with 65% ee other monodentate ligands in the Pd-catalyzed substitution of 1-phenyl-2-propenyl acetate with dimethylmalonate.

## Introduction

**Scheme 1:**
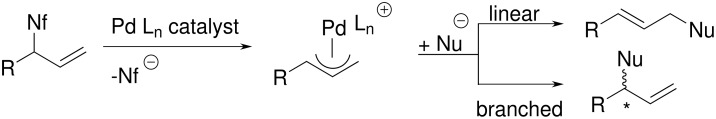
Pd-catalyzed allylic substitution with unsymmetrical substrates (Nu = dimethylmalonate, Nf = OAc).

Palladium catalyzed allylic substitutions provide valuable tools for stereoselective C-C- and C-heteroatom connections [1–2]. The control of regio- and enantioselectivity is challenging, especially with unsymmetrical substrates, e.g. with monoaryl allyl units. According to computational analyses of electronic effects,[3–4] regioselectivity in favor of the branched product is supported at strong donor-substituted (e.g. alkyl, O-alkyl) allylic positions. Frequently employed Pd-catalysts most often favor linear, nonchiral products (Scheme 1).

**Scheme 2:**
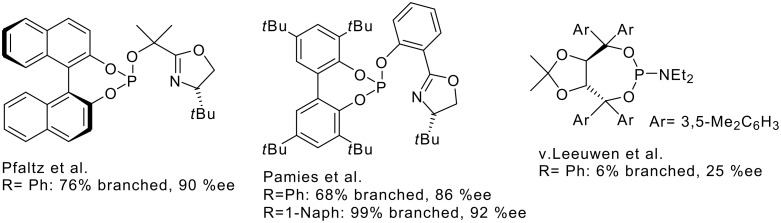
Bidentate P, N-ligands and a monodentate phosphoramidite for Pd-catalyzed allylic substitutions with unsymmetric substrates, cf. Scheme 1.

Pfaltz *et al*. improved the yield of the chiral, branched product by employing electron withdrawing substituents on the P-donor atoms in P, N-oxazoline ligands[5] (Scheme 2) [6]. Such phosphites were thought to favor a more S_N_1-like addition at the substituted, allylic C-atom. High regio- and enantioselectivities were also achieved with biphenylphosphites by Pamies *et al*. (Scheme 2) [7].

Besides bidentate P, N-ligands, monodentate ligands are useful, as was demonstrated successfully by Hayashi *et al*. with the MeO-MOP ligand, yielding 90% branched product with 87% ee for a C-methylated malonate nucleophile and the 4-methoxyphenylallyl substrate [8]. Van Leeuwen's bulky, monodentate TADDOL based phosphoramidite gave rise to intriguing memory effects [28b] and yielded 6% branched product with 25% ee (Scheme 2) [9].

**Scheme 3:**
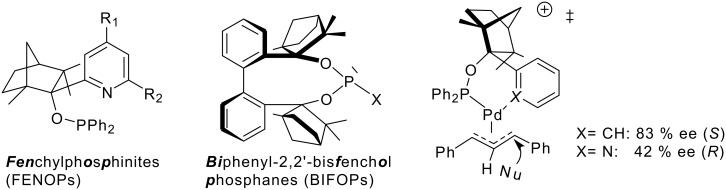
Fenchole-based phosphorus ligands (i.e. FEENOPs and BIFOPs) for Pd-catalyzed allylic substitutions. Pd-π arene or Pd-N coordinations give rise to different enantioselectivitites.

We have recently employed modular, chelating fencholates, [10–14] in enantioselective organozinc catalysts, [15–19] and in chiral *n*-butyllithium aggregates [20–24]. In Pd-catalyzed allylic substitutions of diphenylallyl acetate, fenchyl diphenylphosphinites (FENOPs) with phenyl or anisyl groups favor the *S*-enantiomer, but with a 2-pyridyl unit the *R*-enantiomer was preferred (Scheme 3).[25] According to computational transition structure analyses, these phenyl and anisyl phosphinites are not "monodentate" but form chelate complexes via π-coordination. Biphenyl-2,2'-bisfenchol (BIFOL)[13] was developed as combination of a flexible biaryl axis (as in BINOL) and sterically crowded hydroxy groups (as in TADDOLs). BIFOL based phosphanes (BIFOPs) are sterically highly hindered and were employed in copper-catalyzed 1,4-additions of diethylzinc to 2-cyclohexenone [26].

Here we use a selection of fenchol-based bidentate pyridine FENOP- and monodentate BIFOP-ligands in Pd-catalysts to study allylic substitutions of the challenging 1-phenyl-2-propenyl acetate (Scheme 1, R=Ph) [27–28].

## Results and discussion

**Scheme 4:**
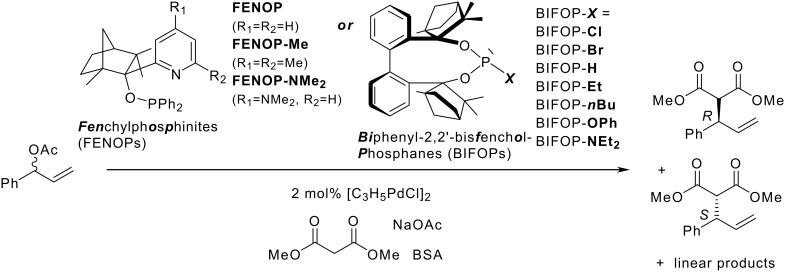
Allylic alkylation of 1-phenyl-2-propenyl acetate by sodium dimethylmalonate (BSA-method) with Pd-FENOP- or Pd-BIFOP- catalysts.

Fenchylphosphinites (FENOPs) and biphenylbisfenchol based phosphorus ligands are all suitable for Pd-catalyzed allylic alkylations of 1-phenyl-2-propenyl acetate (Scheme 4, Table 1, see Supporting Information File 1 for full experimental data).

**Table 1:** FENOP- and BIFOP-Pd-catalysts in enantioselective allylic substitutions of phenylallyacetate by dimethylmalonate.^a)^

Ligand	Linear / *branched* ^b)^	% ee (major enantiomer) ^c)^	% yield ^b)^

**FENOP**	42 / *58*	19 (*R*)	54
**FENOP-Me**	39 / *61*	31 (*R*)	43
**FENOP-NMe_2_**	44 / *56*	37 (*R*)	50

**BIFOP-Cl**	89 / *11*	39 (*S*)	60
**BIFOP-Br**	85 / *15*	37 (*S*)	56
**BIFOP-H**	80 / *20*	65 (*S*)	68
**BIFOP-Et**	85 / *15*	8 (*S*)	70
**BIFOP-*n*Bu**	65 / *35*	5 (*S*)	75
**BIFOP-Oph**	68 / *32*	29 (*S*)	58
**BIFOP-NEt_2_**	52 / *48*	10 (*S*)	52

a) All catalyses were performed in THF, 12 h at -78°C then 24 h at RT with 0.0055 mmol of the ligand, 0.0055 mmol of [Pd(allyl)Cl]_2_ (1 mol% catalyst) and 0.57 mol of 1-phenylallylacetate substrate. b) Linear / branched ratios as well as yields were determined by integration of ^1^H-NMR spectra. c) Enantiomeric excesses (%ee) of the branched products were determined by HPLC (Daicel-OD-H, hexanes / *i*-PrOH = 99/1, 0.55 mi /min., l= 220 nm, t_R_= 16.7 min. (*R*), 17.7 min. (*S*).

**Figure 1:**
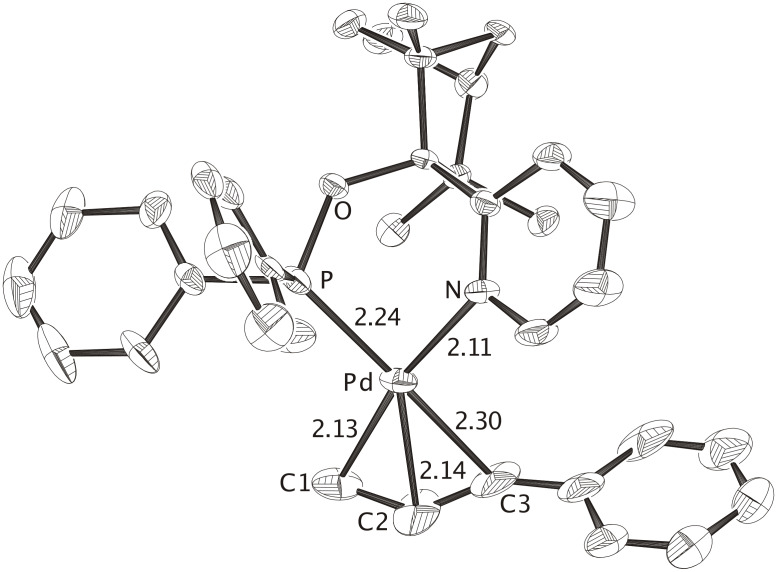
X-ray crystal structure of the cationic complex **Pd-FENOP** (CCDC 299944), the perchlorate counterion and hydrogen atoms are omitted. The allylic phenyl groups is positioned *trans* to phosphorus. In agreement with the the "*trans* rule", C3-Pd is longer then C1-Pd. The nucleophile (i.e. malonate) is expected to attack at C3 yielding the branched product. Distances are given in Angstroms.

**Figure 2:**
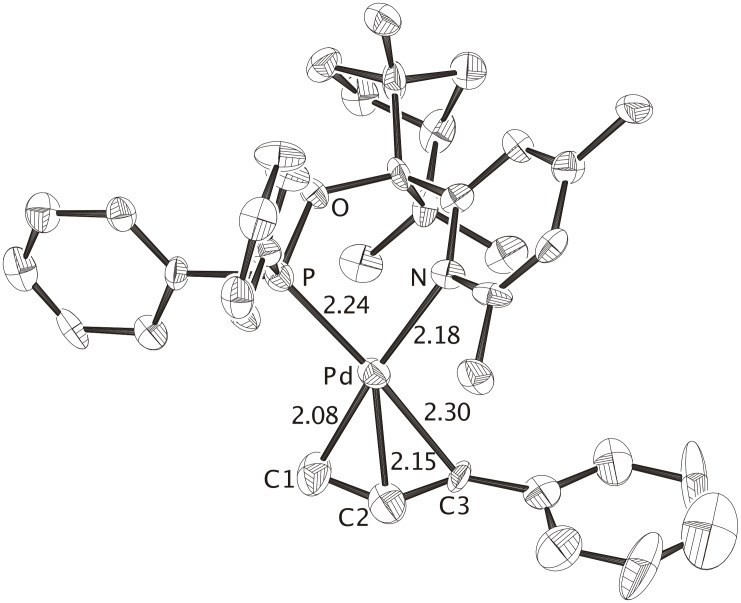
X-ray crystal structure of the cationic complex **Pd-FENOP-Me** (CCDC 600369), the perchlorate counterion and hydrogen atoms are omitted. The allylic phenyl groups is positioned *trans* to phosphorus. In agreement with the "*trans* rule", C3-Pd is longer then C1-Pd. The nucleophile (i.e. malonate) is expected to attack at C3 yielding the branched product. Distances are given in Angstroms.

**Figure 3:**
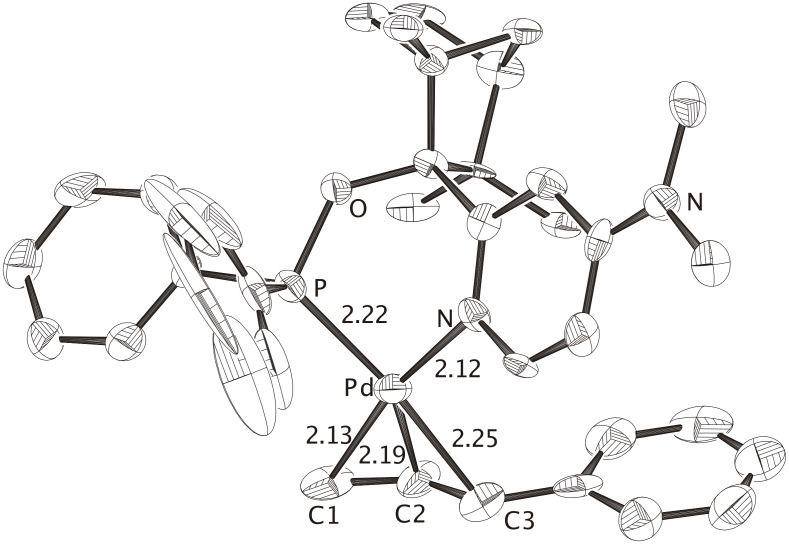
X-ray crystal structure of the cationic complex **Pd-FENOP-NMe_2_** (CCDC 600370), the perchlorate counterion and hydrogen atoms are omitted. The allylic phenyl groups is positioned *trans* to phosphorus. In agreement with the the "*trans* rule", C3-Pd is longer then C1-Pd. The nucleophile (i.e. malonate) is expected to attack at C3 yielding the branched product. The mean values of two independent complexes are given, distances are given in Angstroms.

All three P, N-bidentate FENOP ligands, **FENOP**, **FENOP-Me** and **FENOP-NMe_2_**, favor branched alkylation products (Table 1). This tendency towards formation of chiral, branched products is even apparent from X-ray crystal structure analyses of corresponding Pd-phenylallyl intermediates. All three Pd-allyl complexes, **Pd-FENOP**, **Pd-FENOP-Me** and **Pd-FENOP-NMe_2_** (Figure 1, Figure 2 and Figure 3) exhibit the allylic phenyl group *trans* situated relative to phosphorus. Rather long C3-Pd distances (2.30 Å, 2.30 Å and 2.25 Å) are apparent for these *trans* position in comparison to the shorter C1-Pd bond distances (2.13 Å, 2.08 Å and 2.13 Å, cf. Figure 1, Figure 2 and Figure 3). This differentiation agrees with the "*trans* to phosphorus" rule, [1,29–30] which predicts the attack of the nucleophile (i.e. malonate) at the weakest (longest) C3-Pd bond, yielding preferably the chiral, branched product.

Monodentate BIFOP ligands yield more of the linear alkylation product (Table 1), despite their huge steric demand. Surprisingly, the chloro- and bromophosphites, **BIFOP-Cl** and **BIFOP-Br**, are stable ligands under these reaction conditions: no conversion with nucleophiles (e.g. malonate), as was observed previously with diethylzinc,[26] was found. The ligands were recovered after catalysis. Apparently, the absence of strongly Lewis-acidic electrophiles (Na^+^ vs. Zn^2+^) and the huge steric shielding prevents halide substitutions and **BIFOP-Cl**(**Br**) decompositions.

With regard to enantioselectivities, some monodentate BIFOPs are even superior to the pyridine-phosphinites (FENOPs). While FENOPs favor the *R*-enantiomeric product, the *S*-enantiomer is preferred by all BIFOP ligands. Enantioselectivities increase from **FENOP** with 19% ee to **FENOP-Me** with 31% ee and to **FENOP-NMe_2_** with 37% ee, reflecting the effect of steric demanding and electron donating pyridine groups on enantioselectivity.

The surprisingly stable halogen phosphites **BIFOP-Cl** and **BIFOP-Br** yield even higher enantioselectivities (39% and 37% ee) than the corresponding phosphite **BIFOP-OPh** or the phosphoramidite **BIFOP-NEt_2_** (10% and 29% ee, Table 1). To our knowledge, this is the first successful application of halogen phosphites as ligands in enantioselective catalysis [26]. The highest enantioselectivity however is achieved with the P-H phosphonite **BIFOP-H** (65% ee, Table 1). As in copper-catalyzed 1,4-additions of diethylzinc to cyclohexenone,[26] the small steric hindrance of the hydrido-substituent and the shielding by the chiral bis-fenchane cavity provide the best combination among the tested BIFOPs for the P-H phosphonite **BIFOP-H**.

**Figure 4:**
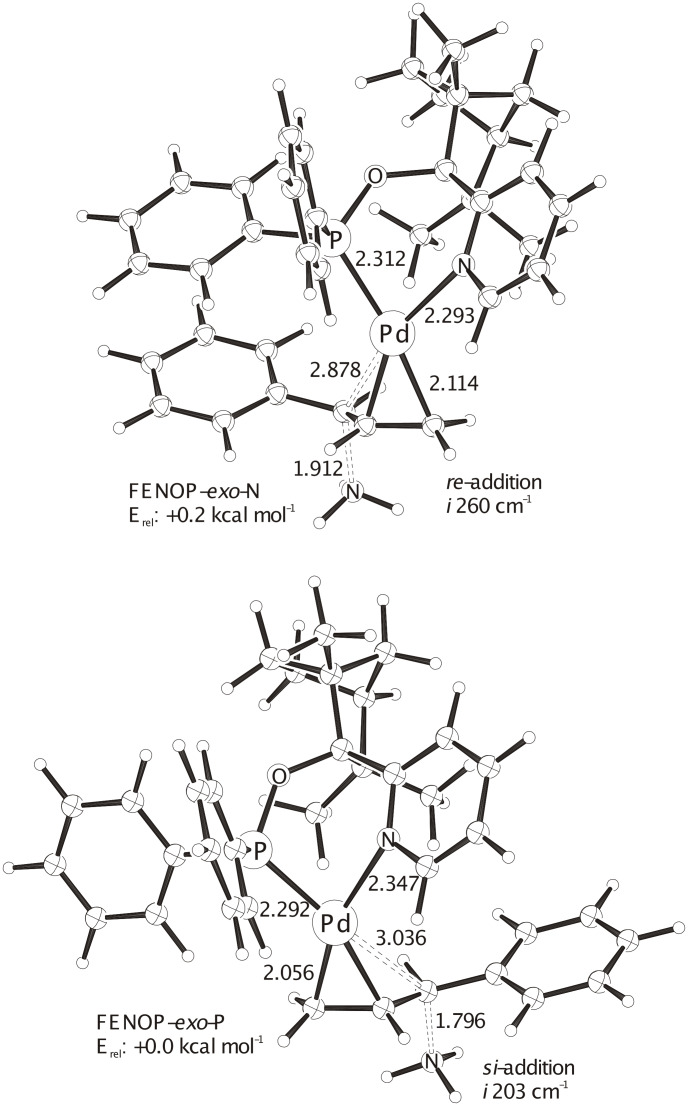
The two most stable ONIOM(B3LYP/SDD(+ECP) (Pd) /6-31G* (C, H, O, N, P) : UFF) optimized transition structures with **FENOP**. ZPE (unscaled) corrected total extrapolated energies: FENOP-*exo*-N (*re*): -1236.56193 H, FENOP-*exo*-P (*si*): -1236.56221 H. The by 0.2 kcal mol^-1^ slightly preferred *si*-addition of the NH_3_ model nucleophile corresponds to the experimental *R*-alkylation product.

**Figure 5:**
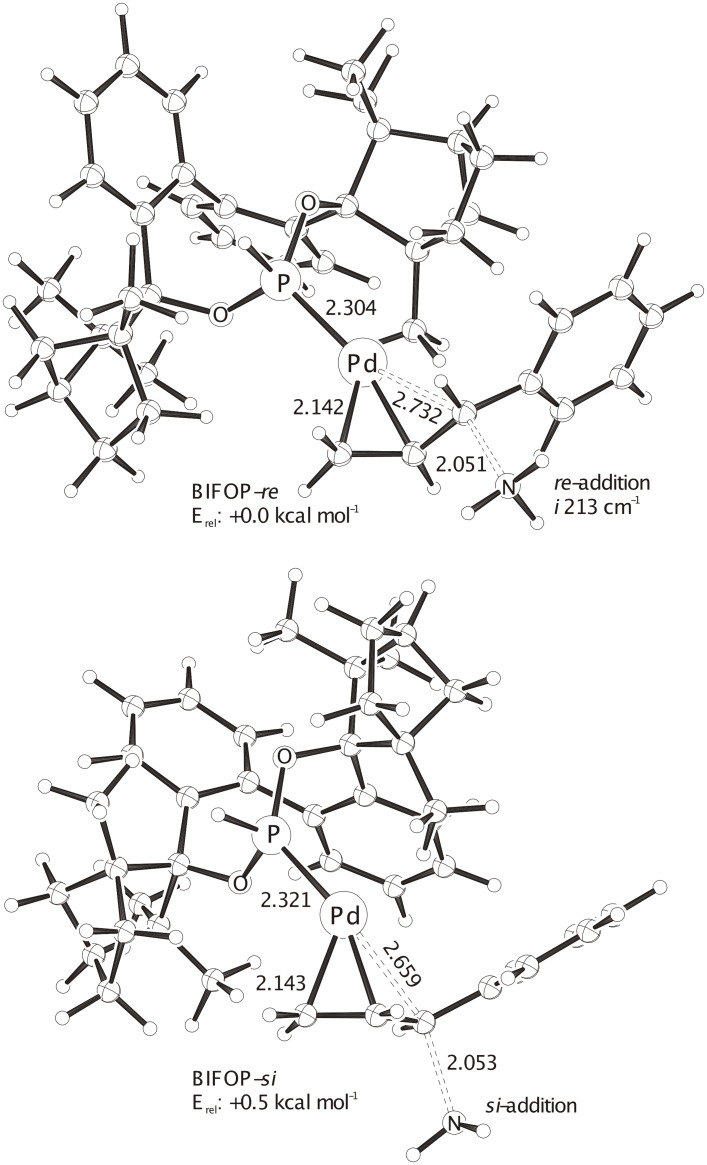
The two most stable ONIOM(B3LYP/SDD(+ECP) (Pd) /6-31G* (C, H, O, N, P) : UFF) optimized transition structures with **BIFOP-H**, due to systematic conformational analysis (60° rotations at P-Pd). ZPE (unscaled) corrected total extrapolated energies: **BIFOP-H-*re***: -1025.01553 H, **BIFOP-H-*si***: -1025.01466 H. The by 0.5 kcal mol^-1^ slightly preferred *re*-addition of the NH_3_ model nucleophile corresponds to the experimental *S*-alkylation product.

Computational transition structure analyses of allylic substitutions with ammonia mimicking the malonate nucleophile help to understand origins of enantioselectivities,[31–34] as we have shown recently for Pd-FENOP catalysts with the diphenyl allyl substrate [25]. For the P, N-bidentate pyridyl **FENOP** system, an *exo* allyl arrangement and a *trans* to phosphorus addition of the nukleophile is slightly preferred (cf. the two most stable transition state in Figure 4). This favored *Si*-addition of the nucleophile explains the experimentally observed formation of the *R*-alkylation product (Table 1). Systematic conformational analyses of transition structures with **BIFOP-H** in allylic substitutions yields **BIFOP-H-*Re*** as the most stable transition structure. Its *Re*-addition of the NH_3_-nucleophile is slightly more favored than the *Si*-addition in the competing transition structure **BIFOP-H-*Si*** (Figure 5). This agrees with the experimentally observed formation of the *S*-alkylation product with BIFOP-ligands (Table 1).

## Conclusion

Besides P, N-bidentate FENOP ligands, monodentate BIFOP ligands can be employed successfully in Pd-catalyzed allylic substitution of 1-phenyl-2-propenyl acetate with dimethylmalonate. Surprisingly, the halogen phosphites **BIFOP-Cl** and **BIFOP-Br** are stable towards nucleophiles under catalysis conditions, apparently due to absence of strongly Lewis-acidic cations and the large steric shielding of the phosphorus-halogen functions. With respect to enantioselectivities, the P-H phosphonite **BIFOP-H** is clearly superior and reaches 65% ee, a rather high selectivity for a monodentate ligand.

## Supporting Information

File 1 contains all experimental data
