# Supplementary material for: A superior P-H phosphonite: Asymmetric allylic substitutions with fenchol-based palladium catalysts
Source: Beilstein J Org Chem. 2006 Mar 30;2:7. doi: 10.1186/1860-5397-2-7 (PMC1479457; doi:10.1186/1860-5397-2-7)
Supplement: File 1 — contains all experimental data [file Beilstein_J_Org_Chem-02-07-s001.pdf]

Experimental Section

General

The reactions were carried out under argon atmosphere (Schlenk and needle-septum techniques) with dried and degassed solvents. X-ray crystal analyses were performed on a Bruker Smart CCD diffractometer with Mo K_{α}-radiation, NMR spectra were recorded on a Bruker AMX300, IR spectra on a Bruker Equinox 55 FT-IR spectrometer and optical rotations on a Perkin Elmer P241 machine. GC analyses were carried out on a Chrompack (CP9001). Crystallographic data (excluding structure factors) for the structures reported in this paper have been deposited with the Cambridge Crystallographic Data Center. Copies of the data can be obtained free of charge on application to CCDC, 12 Union Road, Cambridge CB2 1EZ, UK (Fax: + 44-1223/336-033; E-mail: deposit@ccdc.cam.ac.uk).

Syntheses and characterizations of all BIFOP-ligands have been described recently.[26] The synthesis and characterization of **FENOP** has been described previously.[25] Racemic 1-phenylallyl alcohol was synthesized from benzaldehyde and vinyl magnesium bromide.[34]

Synthesis and characterization of FENOP-Me

a) 2-Bromo-4,6-dimethylpyridine: 62.3 g (0.51 mol) of 2-amino-4,6-dimethylpyridine were reacted according to a general synthesis of bromopridines via diazotation with bromine.[35] Purification via distillation yielded 79.7 g (84.2 %) of the product as yellow oil. Bp.: 95 °C (11 mbar), ^{1}H-NMR ($CDCl_3$), δ: 2.27 (s, 3H, CH3); 2.48 (s, 3H, CH3); 6.92 (s, 1H, H5); 7.12 (s, 1H, H3). MS (EI), m/z [%]: 186 (M^+).

b) (1*R*,2*R*,4*S*)-2-(4,6-Dimethylpyridine-2-yl)-1,3,3-trimethyl-bicyclo[2.2.1]heptan-2-ol: 25.0 g (0.13 mol) of 2-bromo-4,6-dimethylpyridine were lithiated (2 h , -78°C) via bromine/lithium exchange and reacted with (-)-fenchone according to published procedures.[25,36,37] Purification via distillation yielded 24.8 g (71.1 %). Bp.: 127 °C ($1*10^{-3}$ mbar), Mp.: 64-65 °C, $[\alpha]_D$ = -43.5, elemental analysis ($C_{17}H_{25}NO$, M = 259.39 g/mol):calc.: C 78.72 %, H 9.71 %, N 5.40 %, found: C 78.66 %, H 9.68 %, N 5.47 %, ^{1}H-NMR ($CDCl_3$), δ: 0.48 (s, 3H, CH3); 0.99 (s, 3H, CH3); 1.01 (s, 3H, CH3); 1.15 (m, 1H, 6-exo); 1.38 (m, 1H, 7-anti); 1.50 (m, 1H, 5-exo); 1.80 (m, 1H, CH); 1.88 (m, 1H, 5-endo); 2.26 (m, 1H, 7-syn); 2.35 (m, 1H, 6-endo); 2.36 (s, 3H, PyrCH3); 2.48 (s, 3H, PyrCH3); 6.26 (s, 1H, OH); 6.84 (s, 1H, Har); 7.13 (s, 1H, Har); ^{13}C{1H}-NMR ($CDCl_3$), δ: 17.0 (CH3); 21.1 (PyrCH3); 22.1 (CH3); 23.8 (PyrCH3); 24.2 (CH2); 29.1 (CH3); 32.3 (CH2); 41.8 (CH2); 45.6 (Cq); 48.7 (CH); 51.5 (Cq); 83.0 (Cq); 120.8 (Car); 121.6 (Car),

145.9 (Car/q); 154.7 (Car/q); 160.9 (Car/q). IR (KBr, cm^{-1}): 3381 (OH, m), 3010 (Caromat.-H, w), 2990-2870 (Calkyl-H, m); MS (EI), m/z [%]: 259 (M^+).

c) **FENOP-Me** (1*R*,2*R*,4*S*)-2-(4,6-dimethylpyridine-2-yl)-1,3,3-trimethyl-bicyclo[2.2.1]hept-2-yl-diphenylphosphinite): 5.0 g (0.019 mol) of the above synthesized 4,6-dimethypyridylfenchol were reacted in 100 ml THF with diphenyl chlorophosphane according to a published procedure.[25] The yielded crude, yellow oil was diluted with little methanol (ca. 3 ml) and stirred at room temperature. The resulting white precipitate was washed with cold (-20 °C) methanol and dried in vacuum, yielding 5.6 g (66.4 %) of **FENOP-Me**. Mp.: 110 °C, $[\alpha]_D$ = -91.4, elemental analysis ($C_{29}H_{34}NOP$, M = 443.56 g/mol): calc: C 78.53 %, H 7.73 %, N 3.16 %,P 6.98 %, found: C 78.27 %, H 7.54 %, N 3.34 %, P 7.22 %. ^{1}H-NMR ($CDCl_3$), δ: 0.34 (s, 3H, CH3); 0.77 (m, 1H, 6-exo); 1.12 (m, 1H, 7-anti) 1.34 (s, 3H, CH3, m, 1H, 5-exo); 1.50 (s, 3H, CH3); 1.67 (m, 1H, CH, m, 1H, 5-endo, m, 1H, 7-syn); 1.79 (s, 3H, PyrCH3); 2.34 (s, 3H, PyrCH3); 2.78 (m, 1H, 6-endo); 6.65 (s, 1H, Har); 6.72 (s, 1H, Har); 7.27 (m, 6H, Har); 7.64 (m, 4Har). ^{13}C{^{1}H, ^{31}P}-NMR (CDCl3), δ: 18.3 (CH3); 18.5 (PyrCH3); 20.7 (PyrCH3); 22.8 (CH2); 24.9 (CH3); 29.3 (CH3); 33.1 (CH2); 43.0 (CH2); 48.4 (Cq); 48.7 (CH); 54.0 (Cq); 94.3 (Cq); 122.0 (Car); 125.1 (Car), 128.0 (Car); 128.4 (Car/q); 129.0 (Car/q); 129.1 (Car); 129.4 (Car); 130.7 (Car); 131.0 (Car); 143.0 (Car); 145.7 (Car/q); 146.6 (Car/q); 170.5 (Car/q). ^{31}P{1H}-NMR (CDCl3), δ: 89.8 (s). IR (KBr, cm^{-1}): 3020 (Caromat.-H, w), 2995-2880 (Calkyl-H, m), MS (FAB+), m/z [%]: 443 (M^+).

Synthesis and characterization of FENOP-NMe$_2$

(1*R*,2*R*,4*S*)-2- (4-N,N-dimethylaminopyridine-2-yl)- 1,3,3-tri- methylbicyclo[2.2.1]heptan-2- ol: 8.5 g (0.04 mol) of 2-bromo dimethylamino pyridine were lithiated (1 h at -78°C) via bromine/lithium exchange and reacted with (-)-fenchone according to published procedures.[25,36] (-)-Fenchone was added subsequently and the mixture was stirred for 2h at -30°C. Hydrolytic workup and recrystallization from diethylether yielded 10.5 g (95.4 %). Mp.: 140 °C, $[\alpha]_D$ = -11.3, elemental analysis ($C_{17}H_{26}NO_2$, M = 274.40): calc.: C 74.41 %, H 9.55 %, N 10.21 %, found: C 74.43 %, H 9.55 %, N 10.10 %, ^{1}H-NMR ($CDCl_3$), δ: 0.42 (s, 3H, CH3); 0.89 (s, 3H, CH3); 0.96 (s, 3H, CH3); 1.13 (m, 1H, 6-exo); 1.35 (m, 1H, 7-anti); 1.42 (m, 1H, 5-exo); 1.75 (m, 1H, CH); 1.84 (m, 1H, 5-endo); 2.18 (m, 1H, 7-syn); 2.25 (m, 1H, 6-endo); 2.92 (s, 6H, N(CH3)2); 6.10 (s, 1H, OH); 6.30 (dd, 3J = 5.9 Hz, 4J = 2.5 Hz, 1H, Har); 6.60 (d, 4J = 2.5 Hz, 1H, Har); 7.91 (d, 3J = 5.9 Hz, 1H, Har). ^{13}C{^{1}H}-NMR ($CDCl_3$), δ:17.5 (CH3); 22.3 (CH3); 24.4 (CH2); 29.3 (CH3); 32.7 (CH2); 39.3 (N(CH3)2); 42.2 (CH2); 45.7 (Cq); 48.9 (CH); 51.6 (Cq); 83.5 (Cq); 105.1 (Car); 105.8 (Car), 146.4 (Car); 153.7 (Car/q); 162.0 (Car/q). IR (KBr,

cm^{-1}): 3315 (OH, m), 3005 (Caromat.-H, w), 2980-2870 (Calkyl-H, m), MS (EI), m/z [%]: 274 (M+).

FENOP-NMe$_2$ (1*R*,2*R*,4*S*)-2-(4-N,N-Dimethylaminopyridine-2-yl)-1,3,3-trimethyl-bicyclo-[2.2.1]hept-2-yl-diphenylphosphinite): 6.0 g (0.022 mol) of the above synthesized N,N-dimethylaminopyridyl fenchol were reacted in 100 ml THF with diphenyl chlorophosphane according to a published procedure.[25] The yielded crude, yellow oil was diluted with methanol (ca. 10 ml) and was stirred in an ultrasonic bath for ca. 10 min. until a crystalline precipitate formed. The resulting white precipitate was washed with cold (-20 °C) methanol and dried in vacuum, yielding 8.6 g (85.4 %) of **FENOP-NMe$_2$**. Mp.: 136-137 °C, $[\alpha]_D$: = -22.1, elemental analysis ($C_{29}H_{35}N_2OP$, M = 458.57 g/mol):calc.: C 75.96 %, H 7.69 %, N 6.11 %, P 6.75 %, found: C 75.70 %, H 7.78 %, N 5.95 %, P 6.49 %, ^{1}H-NMR ($CDCl_3$), δ: 0.55 (s, 3H, CH3); 0.72 (m, 1H, 6-exo); 1.15 (m, 1H, 7-anti) 1.41 (s, 3H, CH3, m, 1H, 5-exo); 1.55 (s, 3H, CH3); 1.68 (m, 1H, CH, m, 1H, 5-endo, m, 1H, 7-syn); 2.35 (s, 6H, N(CH3)2); 2.91 (m, 1H, 6-endo); 6.28 (m, 1H, Har); 6.45 (m, 1H, Har); 7.20-7.35 (m, 6H, Har); 7.60-7.75 (m, 4Har); 8.18 (m, 1H, Har). $^{13}C\{^1H, ^{31}P\}$-NMR ($CDCl_3$), δ: 18.6 (CH3); 22.9 (CH2); 24.8 (CH3); 28.8 (CH3); 33.0 (CH2); 38.4 (N(CH3)2); 43.2 (CH2); 48.7 (CH); 54.1 (Cq); 58.4 (Cq); 94.8 (Cq); 105.0 (Car); 106.9 (Car), 127.3 (Car); 127.5 (Car/q); 127.7 (Car/q); 128.8 (Car); 129.1 (Car); 129.4 (Car); 130.0 (Car); 131.8 (Car); 147. (Car); 154.0 (Car/q); 162.8 (Car/q). $^{31}P\{^1H\}$-NMR ($CDCl_3$), δ: 87.9 (s), IR (KBr, cm^{-1}): 3051 (Caromat.-H, w), 2980-2890 (Calkyl-H, m), MS (FAB+), m/z [%]: 458 (M^+).

Syntheses and characterizations of FENOP-Pd phenylallyl complexes

General synthetic procedure:

The FENOP- or the BIFOP-ligands (each 0.09 mmol) and palladium allyl chloride (25.9 mg, 0.05 mmol, $C_9H_9PdCl)_2$ were dissolved in 1 ml of acetone and stirred for 1h at room temperature. Lithium perchlorate (80.3 mg, 0.5 mmol) in 1 ml of acetone was added to the yellow solution. Stirring for further 30 min. and evaporation of acetone in vacuum yields a yellowish solid, which was dissolved in 1 ml of methanol. Water (~ 3 ml) is added until no more precipitate is formed. After filtration via syringe the precipitate was dried in vacuum. Dissolving of the yellow precipitate in a hot toluene/diethylether mixture and slow cooling yields single crystals of the FENOP palladium phenylallyl perclorate complexes.

Analytical data of **Pd-FENOP**: Mp.: 165 °C (decomposition), ^{1}H-NMR ($CDCl_3$), δ: 0.50 (s, 3H, CH3); 1.10 (s, 3H, CH3); 1.32-1.40 (m, 1H, 6-exo; m, 1H, 7-anti; m, 1H, 5-exo); 1.75 (s, 3H, CH3); 1.80 (m, 1H, CH; m, 1H, 5-endo), 2.05 (m, 1H, 7-syn), 2.25 (m, 1H, Hallyl.), 2.55 (m, 1H, 6-endo); 2.95 (m, 1H, Hallyl.); 5.12 (m, 1H, Hallyl.), 6.45 (m, 1H, Hallyl.), 6.80-7.80 (m, 18H, Har); 9.40 (m, 1H, Har). ^{13}C{^{1}H, ^{31}P}-NMR ($CDCl_3$), δ: 18.6 (CH3); 22.7 (CH3); 24.8 (CH2); 29.4 (CH3); 32.7 (CH2); 43.1 (CH2); 48.7 (CHq); 48.6 (CH); 53.7 (CHq); 55.6 (CH2(allyl/cis-P)); 94.0 (CHq); 121.1 (Car); 124.2 (Car);128.1 (CHallyl/trans-P); 128.2 (Car/q); 128.4 (Car/q); 128.9 (Car); 129.1 (Car); 129.3 (Car); 130.7 (CHallyl/zentral); 131.3 (Car); 131.5 (Car); 131.7 (Car); 132.8 (Car); 133.7 (Car); 134.7 (Car/q); 143.2 (Car); 145.2 (Car); 147.1 (Car); 163.0 (Car/q). ^{31}P{^{1}H}-NMR ($CDCl_3$), δ: 120.5 (s).

X-ray crystal data of **Pd-FENOP** (CCDC 299944): $C_{36}H_{39}ClNO_5PPd$; M = 738.50; space group $P6_3$; hexagonal; a = 27.8850(2) Å, b= 27.8850(2) Å, c= 9.8813(2) Å, V = 6654.05(15) Å^3; Z = 6; T = 200(2) K; μ= 0.547 mm^{-1}; reflections total: 36123, unique: 3570, observed: 2599 (I >2σ(I)); R1 = 0.057, wR2 = 0.145; GOF = 1.07.

Analytical data of **Pd-FENOP-Me**:

Mp.: 187°C, ^{31}P-NMR ($CDCl_3$) δ: 126.76, ^{13}C-NMR ($CDCl_3$) δ: 10.41, 13.86, 14.22, 14.78, 17.89, 18.01, 18.13, 18.78, 20.55, 22.47, 22.99, 27.67, 27.54, 37.84, 28.19, 28.54, 30.74, 31.55, 37.79, 44.41, 79.77, 80.03, 80.34, 80.92, 109.31, 125.80, 126.46, 126.75, 127.39, 130.52, 131.55, 132.28, 132.76, 137.37, 141.16, 142.46, 144.65, 144.96, 145.24, 384. ^{1}H-NMR (CDCl3), δ: 0.50 (s, 3H,); 1.10 (s, 3H,); 1.32-1.40 (m, 1H, 6-*exo;* m, 2H,); 1.75 (s, 3H,); 1.80 (m, 4H), 2.05 (m, 4H,), 2.25 (m, 2H2.55 (m, 1H); 2.95 (m, 1H), 5.12 (m, 1H6.45 (m, 1H,), 6.80-7.80 (m, 18H); 9.40 (m, 1H).

X-ray crystal data of **Pd-FENOP-Me** (CCDC 600369): $C_{42}H_{53}O_6ClNPPd$ ($C_{38}H_{43}NOPPd$,$C_4H_{10}O$,ClO_4); M = 840.67; space group $P4_3$; tetragonal; a = 10.212(2) Å, b= 10.212 (2) Å, c= 38.578 (7) Å, V = 4023 (1) Å^3; Z = 4; T = 100 K; μ = 0.614 mm^{-1}; reflections total: 7707, unique: 5273, observed: 3089 (I >2σ(I)); R1 = 0.0697, wR2 = 0.1289; GOF = 1.023.

Analytical data of **Pd-FENOP-NMe$_2$**:

Mp.: 201.4°C (decomposition), ^{31}P-NMR ($CDCl_3$): 122.65, ^{13}C-NMR ($CDCl_3$), δ: 13.41, 13.88, 14.22, 14.78, 17.89, 18.01, 18.13, 18.78, 22.47, 22.99, 27.67, 27.54, 37.84, 28.19, 28.54, 30.65, 31.55, 37.89, 44.41, 79.76, 80.03, 80.34, 80.92, 109.31, 125.80, 126.46, 125.75, 126.59, 130.52,

131.55, 132.28, 132.76, 137.37, 141.16, 142.46, 143.65. ^{1}H-NMR ($CDCl_3$), δ: 0.46 (s, 3H,); 1.03 (s, 3H,); 1.32-1.40 (m, 6H), 1.75 (m, 6H,); 1.89 (m, 4H), 2.15 (m, 4H,), 2.26 (m, 2H), 2.55 (m, 1H); 2.95 (m, 1H), 5.12 (m, 1H),6.45 (m, 1H), 6.52-7.60 (m, 18H); 9.53 (m, 1H).

X-ray crystal data of **Pd-FENOP-NMe$_2$** (CCDC 600370): $C_{45}H_{51}ClN_2O_5PPd$ (C_7H_8,$C_{38}H_{43}N_2OPPd$, ClO_4); M = 1729.27; space group P2$_1$; monoclinic; a = 13.662 (3) Å, b= 15.262 (2) Å, c= 19.576 (3) Å, V = 4069(1) Å^3; Z = 4; T = 100 K; μ = 0.609 mm^{-1}; reflections total: 18704, unique: 14510, observed: 7406 (I >2σ(I)); R1 = 0.0609, wR2 = 0.0959; GOF = 0.884.

General procedure for FENOP- and BIFOP- Pd-catalyzed allylic substitutions

In 1 ml of CH_2Cl_2 4.2 mg of $[PdCl(C_3H_5)]_2$ (0.011 mmol) and 0.022 mmol of the ligand (FENOP or BIFOP) were solved. The yellow solution is stirred for 30 min. at room temperature, then 140 µl 1-phenyl-2-propenyl acetate were added and the mixture was stirred for another 30 min. and was then cooled to -20°C. 280 µl dimethylmalonate, 560 µl *N,O*-bis(trimethyl)acetamide (BSA) and few crystals of potassium acetate were added.[38] The mixture is stirred for 24h at -20°C, was then hydrolyzed with water (NH_4Cl). The organic phase was filtered on silica gel. The enantiomeric excess was determined by HPLC on a DAICEL-OD-H column, hexanes/isopropanol= 99/1, l=254 nm, t_R= 29.9 min (R), 32.5 min (S).

Computational Section

All computed structures were fully optimized using GAUSSIAN 03.[39] For ONIOM[40,41,42] (B3LYP[43,44,45]:UFF[46]) computations, hydrogen atoms were used as linkers between the layers. All structures were analyzed by frequency computations, the imaginary frequencies correspond to C-N bond formations. The SDD/ECP basis set[47] was employed for Pd, the 6-31G* standard basis sets[48] were used for all other atoms.
